# Effects of Growth Media on the Diversity of Culturable Fungi from Lichens

**DOI:** 10.3390/molecules22050824

**Published:** 2017-05-17

**Authors:** Lucia Muggia, Theodora Kopun, Martin Grube

**Affiliations:** 1 Department of Life Sciences, University of Trieste, via Giorgieri 10, 34127 Trieste, Italy; 2 Institute of Plant Science, Karl-Franzens University of Graz, Holteigasse 6, 8010 Graz, Austria; theodora.kopun@uni-graz.at (T.K.); martin.grube@uni-graz.at (M.G.)

**Keywords:** Dothideomycetes, Eurotiomycetes, Leotiomycetes, nuclear ribosomal subunits DNA, nutrients, Sordariomycetes

## Abstract

Microscopic and molecular studies suggest that lichen symbioses contain a plethora of associated fungi. These are potential producers of novel bioactive compounds, but strains isolated on standard media usually represent only a minor subset of these fungi. By using various in vitro growth conditions we are able to modulate and extend the fraction of culturable lichen-associated fungi. We observed that the presence of iron, glucose, magnesium and potassium in growth media is essential for the successful isolation of members from different taxonomic groups. According to sequence data, most isolates besides the lichen mycobionts belong to the classes Dothideomycetes and Eurotiomycetes. With our approach we can further explore the hidden fungal diversity in lichens to assist in the search of novel compounds.

## 1. Introduction

Lichens are self-sustaining symbiotic associations of specialized fungi (the mycobionts), and green algae or cyanobacteria (the photobionts), which are located extracellularly within a matrix of fungal hyphae and from which the fungi derive carbon nutrition [1]. Lichens are characterized by a specific structure, the lichen thallus, which is typically determined by the mycobiont (hence, lichens are classified according to the mycobiont). The classic concept of lichens as a dual partnership has been emended recently, since other microorganisms such as bacteria and additional fungi are regularly present in the thalli [2,3,4,5,6,7,8,9,10]. There is evidence from culture-independent methods that associated bacterial communities potentially influence the fitness of the lichen thallus [11,12]. 

Such work still needs to be accomplished with the fungal associates of lichens, but it is known that numerous lichenicolous fungi can modify the morphology of their hosts [13,14]. The biological effects of lichen-inhabiting (lichenicolous) fungi range from degradation to hypertrophication (formation of fungal galls) of their hosts. Unfortunately, nothing is yet known about the regulatory processes and effective molecules that mediate these fungal interactions. In a pioneering attempt, Hawksworth et al. [15] used lichen thalli and thin-layer chromatography to directly detect compounds possibly originating from lichen-invading fungi. This approach provided a first insight into compound patterns involved in fungal interactions, but these are restricted to bulk compound in the thallus, and may overlook regulatory molecules acting at low concentration. We think that improvement and standardization of culturing conditions are needed to promote discovery of compounds produced by lichen-inhabiting fungi and the further study of their bioactive potential. The search for novel compounds from lichenicolous fungi is not just a matter of mere academic curiosity, but can be of broader pharmaceutical interest. A recent review by Kellogg and Raja [16] lists already 140 novel secondary metabolites from cultured lichenicolous fungi that have been found recently, including information about bioactivity as far as it is known today. For the complexity and diversity of lichenicolous life strategies, lichens are a particularly rich source of yet to be discovered fungi and compounds.

However, some technical issues remain to be solved with complex symbiotic systems, such as lichens. Externally visible fungi, or those which produce phenotypic symptoms on their hosts, are not necessarily the same fungi that can be retrieved easily in culture. In fact, symptomless fungi residing or growing in lichens are very common [3]. The biological roles and the abundances of these cryptically occurring (=endolichenic) fungi are still unclear. Although numerous fungi have been retrieved by culture-dependent approaches so far [9,17,18], only a few studies reliably assign isolates to visible phenotypes of lichenicolous fungi [19,20,21,22].

Culturing protocols are available since long time for the mycobiont of lichens and these were improved over the past decades [23,24,25,26,27,28,29]. Different media compositions may modulate the growth of the mycobiont and may also lead to different chemical spectra, which quite often differ from those of the native lichens [30,31,32,33,34]. While culturing the mycobionts is thus well established, the isolation of the lichen-associated fungi is not properly explored. Handling of the material prior to isolation of fungi can significantly influence the results. For example, inappropriate handling and post-harvest moulding may introduce fungi not present originally, or limited surface sterilization methods could favor fungi loosely attached to the surface [7,8,9,10,11,12,13,14,15,16,17]. Hardly any study so far has compared media composition for the influence on the growth of the lichen-associated fungi in axenic culture and whether isolates representing different fungal classes could be specifically retrieved using different media. In this study we focused on the cultivation of fungi associated with crustose, epilithic lichens (Appendix A). We argued that these lichens and their inhabitants [9] might have more stable (mineral-dependent) substrate parameters than lichens on organic substrates. 

## 2. Results

### 2.1. Molecular Identification of the Isolated Fungal Strains

We report here the isolation of 92 lichen-associated fungal strains: 67 Eurotiomycetes, 14 Dothideomycetes, eight Leotiomycetes and three Sordariomycetes (Appendix B and Appendix C). These isolates are genetically identified in each of the four fungal classes within the same lineages previously recognized by Muggia et al. [9]. In Eurotiomycetes most of the new isolates represent melanized fungi and are recovered in one major lineage (clade VI, Appendix B)—confirming the recurrent occurrence of this fungus in crustose lichens—found in different lichen species and in multiple thalli of the same lichen species. Additional three, minor lineages (clade IV, V, and VII, Appendix B) also group melanized strains. In Dothideomycetes the new isolates are recovered within Pleosporales in the *Phoma* lineage and in Myriangiales. Leotiomycetes and Sordariomycetes are the least represented among the retrieved strains: only 11 isolates have been identified in addition to those reported by Muggia et al. [9]. The recovered isolates in Leotiomycetes represent two unnamed lineages, the first within the core of the class and the second unresolved at its base (Appendix B). Within the Sordariomycetes the isolated strains mainly belong to the orders Xylariales and Coniochaetales. Only two isolates were identified as Agaricomycetes (not shown). In total 29 isolates of Lecanoromycetes, corresponding to the lichen mycobionts, have also been obtained. Only 10% of the total isolates were green algae (not included in any further analyses). Although we considered an expanded range of culture media, we did not recover any Basidiomycetes isolate. 

### 2.2. Correlation of Fungal Strains with Type of Growth Media 

**Figure 1:**
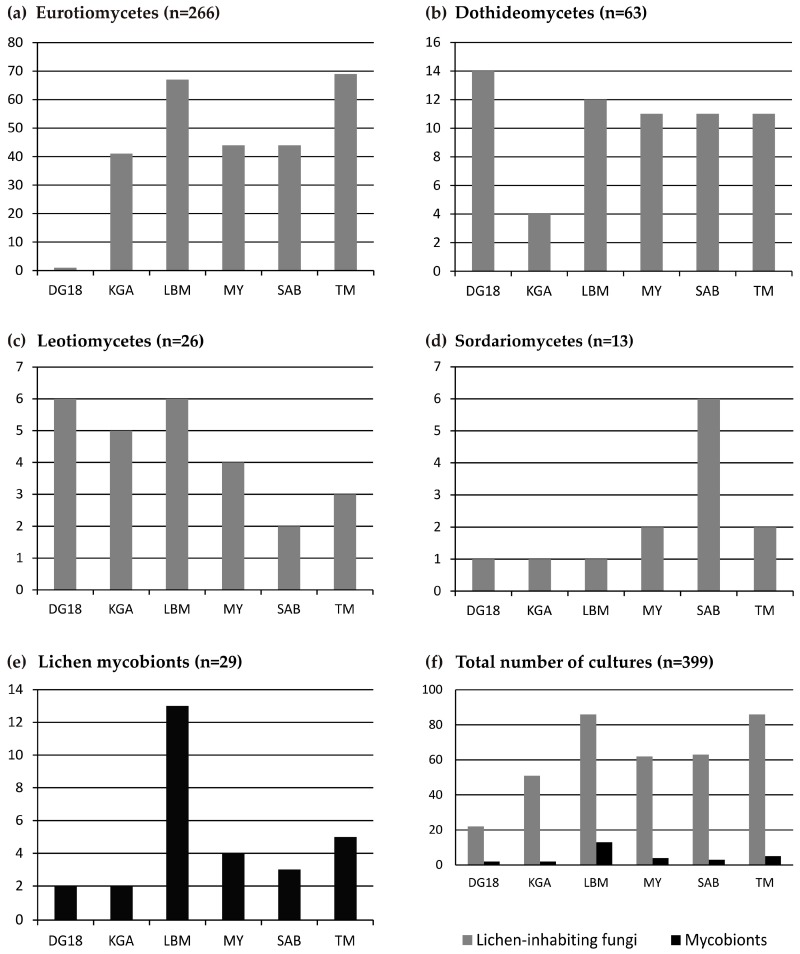
Abundance plots showing the growth preference of the fungal isolates on the six growth media: (**a**) Eurotiomycetes; (**b**) Dothideomycetes; (**c**) Leotiomycetes; (**d**) Sordariomycetes; (**e**) lichen mycobionts; (**f**) total number of lichen-inhabiting fungal isolates compared with isolates of the lichen mycobionts. DG18: Dichloran/Glycerol agar, KGA: PDA Potato/Dextrose agar, LBM: Lilly and Barnet medium, MY: Malt Yeast-extract, SAB: Sabouraud, TM: *Trebouxia* medium.

**Figure 2:**
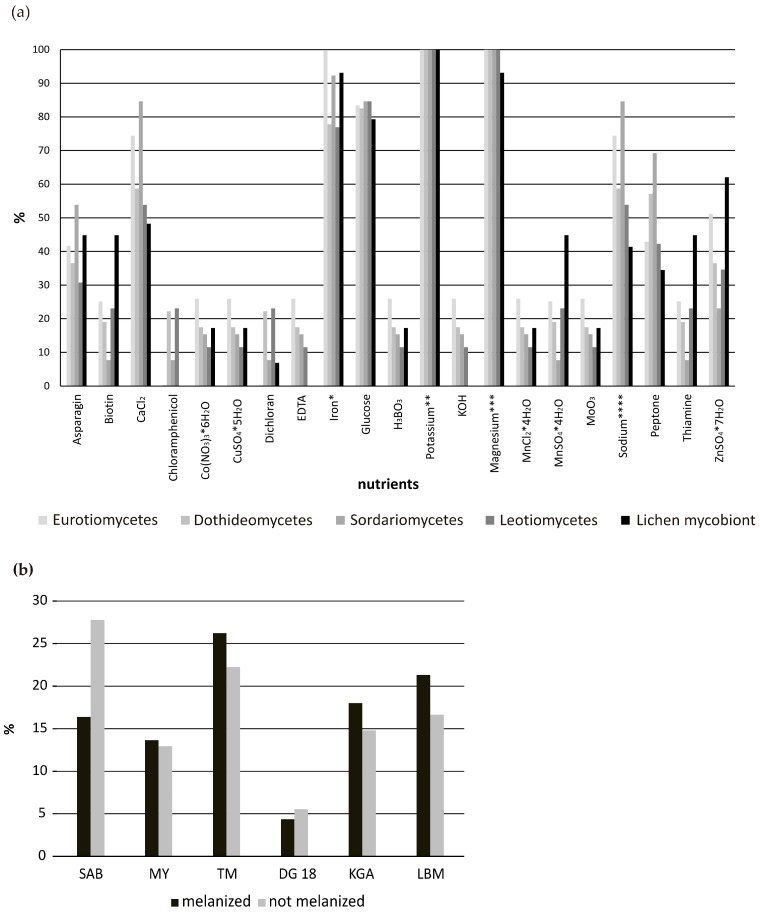
(**a**) Abundance plot showing the growth preference of the five groups of fungal isolates (non-lichenized Eurotiomycetes, Dothideomycetes, Leotiomycetes, Sordariomycetes and lichen mycobionts, mostly Lecanoromycetes) according to the micronutrients present in the culture media. Abundance values are expressed in percentage. The presence of iron, potassium, magnesium and sodium in the medium compounds is summed up as follows: * iron: Fe(NO_3_)_3_·9H_2_O, FeSO_4·_·7H_2_O or iron (from yeast extract); ** potassium: K_2_HPO_4_, KH_2_PO_4_ or potassium (from yeast and malt extract); *** magnesium: MgSO_4_·7H_2_O or magnesium (from yeast and malt extract); **** sodium: NaCl, NaNO_3_; (**b**) Abundance plot comparing the growth preference of melanized and non-melanized fungi on the six culture media (total number of individuals compared *n* = 243). DG18: Dichloran/Glycerol agar, KGA: PDA Potato/Dextrose agar, LBM: Lilly and Barnet medium, MY: Malt Yeast-extract, SAB: Sabouraud, TM: *Trebouxia* medium.

The successful isolation and growth of strains representing the different fungal groups was correlated with the type of medium (Figure 1 and Figure 2). The media differed in the presence of inorganic and organic compounds and were differently enriched by nutrients, such as sugars, metal compounds, amino acids and vitamins (Appendix D). Our analyses include the new isolates here presented and those previously published by Muggia et al. [9] for a total of 399 strains. Eurotiomycetes were mainly isolated on *Trebouxia* Medium (TM) and Lilly & Barnet Medium (LBM), whereas Dichloran/Glycerol agar medium DG18 turned out to be ineffective for their growth (Figure 1a). Dothideomycetes mainly grew on DG18 and LBM (Figure 1b), Leotiomycetes on LBM and DG18 (Figure 1c) and Sordariomycetes on Sabouraud Medium (SAB) (Figure 1d). Lichen mycobionts were isolated mostly on LBM (13 out of 29 isolates) and TM (Figure 1e). In general LBM and TM proved to be the most suitable media for the isolation of a maximum number of fungal taxa, as 52% of the isolates (comprising Eurotiomycetes, Dothideomycetes, Leotiomycetes and lichen mycobionts) grew well on them (Figure 1f). 

We observe that the presence of magnesium, potassium, iron and glucose in growth media is pivotal for the successful isolation of all the fungal classes (Figure 2a). Alternatively, the presence of chloramphenicol in DG18, ethylendiamintetraacetic acid (EDTA) and potassium hydroxide in TM highly reduces the successful isolation of Eurotiomycetes and lichen mycobionts. Zinc and manganese compounds, alternatively, seem to be important for the growth of the lichen mycobionts, whereas asparagine, CaCl_2_, sodium and peptone (majorly in SAB and TM) seem to be important for the growth of Sordariomycetes. Fungal growth in our experiments was not dependent on the pH of the media, as this was kept within a narrow range in all media with values of (5.2–) 5.6 (–6.0).

The great majority of the isolated strains represent melanized fungi belonging to the classes Eurotiomycetes and Dothideomycetes. They were isolated mostly on TM, and this seems to correlate with the abundant presence of metal ions in the medium. Non-melanized fungi, alternatively, have been mostly isolated on SAB. The percentages of melanized and non-melanized fungi isolated on the other four media differ only slightly (Figure 2b).

The environmental samples used as source of fungal isolation are lichens belonging to the class Lecanoromycetes, to which also certain lichenicolous fungi belong. In the environmental samples the morphological determination of lichenicolous fungi revealed the presence of Arthoniomycetes, Dothideomycetes and Eurotiomycetes. Lecanoromycetes are, however, the least obtained isolates (29, genetically identified only as the lichen mycobionts) and the highest number of isolates is represented by Eurotiomycetes (266) and to a lesser extent by Dothideomycetes (representing the lichenicolous and the endolichenic fungi). Arthoniomycetes did not grow on any medium used. The presence of Agaricomycetes, Leotiomycetes and Sordariomycetes in the thalli could be assessed only when the corresponding isolates were genetically identified.

Isolates belonging to Eurotiomycetes, Dothideomycetes, Leotiomycetes and Sordariomycetes developed a mycelium of 5–10 mm in diameter within three months. Isolates corresponding to lichen mycobionts were characterized by a slower growth rate and mycelia of up to 5 mm in diameter could be recovered only one year after the original inoculation. 

## 3. Discussion

To our knowledge, only Vinayaka et al. [35] have compared the influence of three different media on the success rate and systematic bias of endolichenic fungal isolation. The authors recovered 30 taxa, of which the highest number of isolates grew on malt yeast extract (MYA) medium [35]. We have analyzed here six growth media and have recovered the highest number of isolates on LBM. The presence of biotin and thiamin (vitamins B1, B12) in LBM is likely to facilitate the development of the mycelia on the artificial substrate. The highest numbers of melanized isolates are recovered on TM and LBM. It remains nevertheless difficult to compare precise nutrition requirements of the fungal groups because some media ingredients are only sold as extracts, such as potato, malt and yeast extract. The approximate composition of these condiments had to be retrieved from literature or chemical studies of their components. These media are both rich in metal ions, which may enhance the isolation of those fungi which are able to metabolize these compounds for faster growth. 

The diversity of lichen–associated fungi recovered in culture may depend on the type of surface sterilization applied on the thallus and the growth medium used in the initial isolation step. Several protocols for thallus sterilization have been reported in the literature [3,36,37], which apply either washing steps with sodium hypochlorite solutions or ethanol dilutions. In this study thallus fragments were washed with Tween 80 to remove a great part of loosely attached bacteria, with the aim to increase the isolation success of lichen-inhabiting fungi, and cleaning the thalli as much as possible from other spurious particles. The thallus pieces were then smashed in sterile conditions and fragments of less than 0.5 mm in size were inoculated. It should, however, be noticed that lichens do not have a clear separation of external and internal colonization by microorganisms as found in plants, where the cuticula forms a clear border between external and internal microbiota. As microorganisms invade the layers of lichens at variable depths the duration of surface sterilization procedures decreases the number of fungi that can be retrieved. Moreover, the access of sterilization liquids to lichen surfaces is dependent on the microarchitecture and hydrophobicity of lichens. The swelling of hydrated lichens fragments may limit the access of sterilization solutions to thallus fissures (such as cracks between the areoles of crustose lichens). Thus, fungi attached on the surface of these thallus fissures would not be degraded unequivocally by sterilization. In addition, if lichens possess hydrophobic surface structures, this may also prevent access of polar sterilization.

A recent review highlights the potential of lichenicolous fungi as bioresources of novel bioactive compounds [16]. Due to the limited morphological characters and frequent lack of reproductive structures in the cultured mycelia, these strains are hardly identifiable without DNA sequence data. Up to now, in the majority of the cases, the new metabolites are reported from unidentified fungi, which are characterized by strain numbers or morphotypes assignable to hyphomycete genera that lack phylogenetic background [31,38]. The antibacterial compounds lichenicolin A and B, active against Gram-positive bacteria, were isolated from the strain labelled as LL-RB0668 [31]. New heptaketides were isolated from an endolichenic *Corynespora* sp. from *Usnea cavernosa* with cytotoxic activities against cancer cell lines [39]. Furthermore, eight novel metabolites have been characterized from an endolichenic pleosporalean fungus [40]. Some of these fungi were also tested for the production of diverse metabolic patterns on different media or against human pathogenic bacteria for pharmaceutical purposes [38,41]. 

The fungal strains tested so far for their metabolite production mostly derived from epiphytic and terricolous macrolichens, the thalli of which are leaf-like or fruticose (highly three-dimensional), and represent very widespread genera—such as *Aspergillus*, *Chaetomium*, *Penicillium*, *Sporomiella*, *Trichoderma*—of endophytes commonly found as plant pathogens and saprotrophs [41]. In many cases the host lichens are unknown either, which hampers reproducibility of the results. Exceptions are *Aspergillus versicolor*, producing three new anthraquinones derivatives, isolated form the lung lichen *Lobaria retigera* [42], and *Sporomiella irregularis* producing the new xanthone glycoside sporormielloside, isolated from *Usnea mutabilis* [43]. 

Interestingly, many of the isolates obtained here or previously characterized by Muggia et al. [9] from epilithic lichens do not correspond to the genera mentioned above. It seems, therefore, that the growth form of the lichen hosts influences the diversity of the associated fungi: while Eurotiomycetidae, Leotiomycetes and Sordariomycetes are mainly recovered from foliose and fruticose macrolichens [3,7], taxa belonging to Chaetothyriomycetidae and Dothideomycetes are mainly isolated from crustose thalli on rocks [9,44]. Many of these fungi represent new monophyletic lineages, which occur in diverse lichen hosts or share the same host species. The different growth rates that these taxa present in axenic culture might also be correlated with their specificity towards the lichen hosts. Slow growing fungi may be more adapted to the lichens than the ubiquitous strains, which present faster growth rates and may be nutrient-deprived in lichens but proliferate on culture media. Further, it might be speculated that, beside the three dimensionally structure, the air-filled medulla of foliose and fruticose lichens increases the frequency and the duration of condition with limited gas diffusions due to the lack of hydrophobicity. Therefore a longer air-filled medulla in (some but not all) crustose lichens might play a role in the composition of associated fungi.

**Figure 3:**
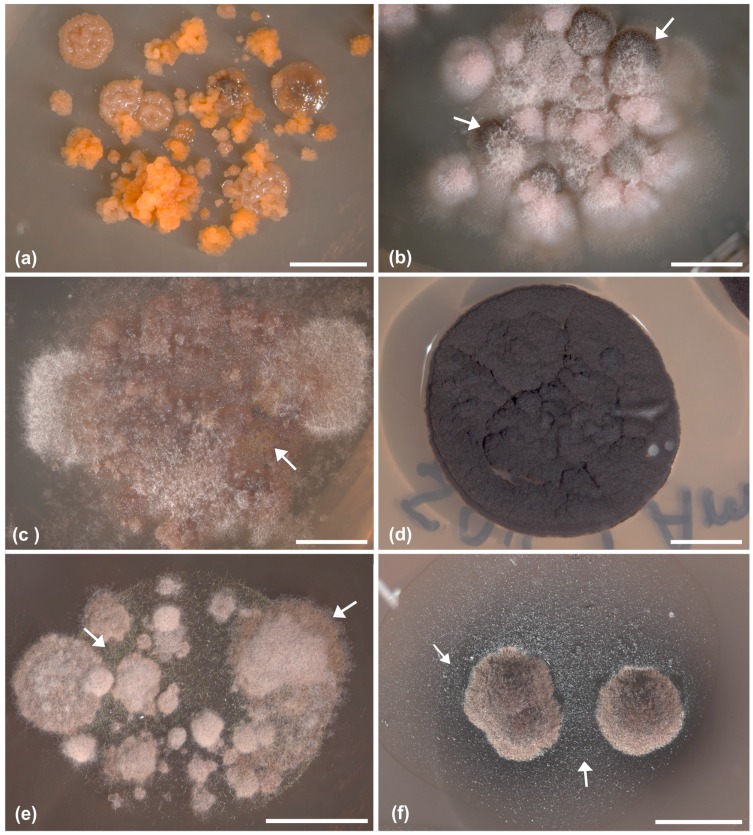
Habit of representative fungal strains which develop peculiar phenotypes of secondary metabolites on diverse culture media (the acronym of medium name and the number of the strain are reported in parentheses): (**a**,**b**,**d**) Eurotiomycetes (Chaetothyriales, A1109, A1165 and A527) fungi (on MY and SAB); (**c**,**e**) Dothideomycetes fungi (on DG18; A1086 and A1168); (**f**) lichen mycobiont *Tephromela atra* (on LBM, A1180). (**a**) Two different strains with diverse phenotypes have grown out from a single inoculum. (**d**) Chaetothyrialean black fungi (A527) have been the most commonly isolated strains from rock inhabiting lichens. Arrows indicates the areas of the mycelia (**b**,**c**,**e**) or of the medium (**f**) where secondary metabolites accumulate and deserve further analyses. Scale bars = (**a**–**d**,**f**) 4 mm, (**e**) 5 mm.

So far, crustose lichens have been seldom considered as sources of lichen-associated fungi, but our results highlight that they harbor a great diversity of lichen-inhabiting fungi, which excrete pigments into the medium (Figure 3) and deserve further chemical characterizations for the exploitation of their metabolic potential. Many of these organisms remain uncultivable [10] because they require the biological context in the lichen species host, which can hardly be provided by in vitro conditions. This may lead to an under-representation of the true organismal diversity in lichens.

With our work we intended to expand the spectrum of cultivable lichenicolous fungi by using a wider range of media conditions. Certainly, this will not lead to a complete inventory of these interesting fungi, but provide means for selective isolation and for better growth of biotechnologically or pharmaceutically interesting fungi. We think that lichen-specific fungi are worth of further investigation for bioactive principles, as these fungi are adapted to live in symbioses with their hosts. As a further step towards uncovering the metabolic potential of lichen-inhabiting fungi, we envision the efficiency of co-culturing of symbionts, which may also involve bacteria as a common component of lichens. Lichen-associated bacteria have already been shown to represent chemically interesting bioresources [45], but their potential effect on a wider range of lichen-derived fungi in co-cultures remains an exciting endeavor for future studies. 

## 4. Materials and Methods 

### 4.1. Sampling and Culture Isolation 

Lichen thalli were sampled in May–July 2012 (Appendix E) on the Koralpe mountain range (Styria, Austria), as described in Fleischhacker et al. [46]) and Muggia et al. [9]. Subsets of thalli visibly infected by 34 species of lichenicolous fungi (Appendix A)—at least 15 samples from each plot—were selected for culture isolation so that we had a total of 190 thalli representing 21 lichen species. We considered only lichens from rocks, majorly with a crust-like growth type. The ratio of crustose to foliose lichens was 9:1. 

The fungal isolation followed the protocol of Yamamoto et al. [47] and is schematically reported in Appendix F. Briefly, approximately 2 mm^2^ fragments of infected lichen thalli were dissected with a sterile razor blade. The fragments were washed three times for 15 min with distilled sterile water, 30 min with 500 μL Tween 80 (diluted 1:10) and finally twice for 15 min with sterile water. As the aim of the study was to isolate lichenicolous fungi and any other fungus residing within the lichen thallus [9,46], the washing steps were performed to remove any spurious organism or particles (bacteria, spores, fragments of fungal hyphae, yeast) loosely attached on the thallus external surface. The washed samples were then grinded in sterile water and tiny thallus fragments were picked and transferred individually into slanted agar tubes. In order to promote the growth of as many different lichen-inhabiting fungi as possible, covering a broad spectrum of fungal growth requirements, we inoculated the dissected fragments on six different media (Appendix D): Lilly & Barnett (LB [48]); *Trebouxia* medium (TM [49]), Malt Yeast-extract (MY [48]), Sabouraud (SAB [50]), Potato Dextrose agar (PDA=KGA, ApplChem A5828), Dichloran/Glycerol agar (DG18 [51]). MY, SAB and PDA are full, organic media containing several micronutrients and are therefore used to characterize a wide variety of fungi and yeasts [52]. LB medium is principally used for the isolation of lichenized fungi, while TM is mainly used for the growth of photobionts, but as it is also rich in carbohydrates it works well also for lichenized and non-lichenized fungi [49]. To cover a broader spectrum in fungal growth requirements we also used DG18, which is a special medium for xerophilic fungi [51]. In total we inoculated 5400 tubes (Appendix F). Five tubes of each medium were inoculated for each sample, resulting in a total of 30 inocula. The tubes were incubated in a growing chamber at 20 °C, with a light-dark regime of 14:10 h, light intensity of 60–100 μmol photons m^−2^s^−1^ and 60% humidity. After three to five months, the inocula reached about 1–3 mm in diameter and it was possible to gain subcultures which were necessary for the isolation of DNA for genetic identification and morphological analyses [9]. The subcultures were set on agar Petri plates using the same growth medium where the inoculum grew successfully; ampicillin was further added to avoid eventual post bacteria contamination. The cultured strains are stored at the University of Graz in the culture collection of the first author LM and are preserved as cryostocks, as cited in Muggia et al. [9]. 

### 4.2. Morphological Analyses 

Morphological and anatomical characters of the cultured strains were analyzed using light microscopy and documented with digital photographs as in Muggia et al. [9]. Analyses and photographs were performed on 10 month to one year old subcultures considering the following characters: form of growth, branching of the hyphae and melanization. Small fragments of the mycelia were taken; squashed sections were mounted in water and studied by light microscopy. All images were acquired with a ZEISSAxioCam MRc5 (Zeiss, Jena, Germany) digital camera fitted to the microscope. Both images of growth habit and hyphae structure [9] were digitally processed using the CombineZM software [53]. The photos were slightly refined in sharpness and color tone with Adobe Photoshop 7.0 (© Adobe System Incorporated, San Jose, CA, USA) and the figures were prepared with CorelDRAW X7 (© Corel Corporation, Ottawa, Canada).

### 4.3. DNA Extraction, Amplification and Sequencing 

The identity of each grown isolate was checked with sequences of at least two genetic markers. Small parts of the sub-cultured fungi were taken, transferred into 1.5 mL reaction tubes containing sterile tungsten beads (Qiagen, Vienna, Austria) for homogenization, frozen and ground using a TissueLyserII (Retsch, Haan, Germany). The DNA was extracted following either the cetyltrimethyl ammonium bromide CTAB protocol of Cubero et al. [54] or using the DNeasy Plant Mini Kit (Qiagen). The industrial kit was used for those most melanized isolates for which the CTAB protocol failed in extracting amplifiable DNA. 

The identity of the cultured fungal strains was studied with sequences of the nuclear large and partial nuclear small ribosomal subunits (nucLSU and nucSSU) and the mitochondrial small ribosomal subunit (mtSSU). Primers and PCR conditions, sequencing and sequence analysis were performed as in Muggia et al. [9]. 

### 4.4. Phylogenetic Analyses for Isolate Identification

We checked the identity of the newly generated sequences with sequences available in the GenBank database by BLAST similarity search [55] and with those generated previously by Muggia et al. [9]. Taxa which most closely matched our sequences for a value not lower than 95% identity and the further most closely related ones (up to 90% identity) were selected for the phylogenetic analyses. As our sequences (Appendix C) showed closest matches with representatives of the classes Eurotiomycetes (particularly in the subclasses Chaetothyriomycetidae), Dothideomycetes, Leotiomycetes and Sordariomycetes, we prepared four different datasets representing each fungal group. The here newly obtained sequences were added to the datasets previously constructed by Muggia et al. [9], which were carefully selected on the base of previous phylogenetic analyses considering the aforementioned classes [56,57,58,59,60,61,62,63,64,65,66,67]. Each dataset represents the widest possible spectrum of taxon diversity, including at least three representative taxa for each different family or order of the four classes (Appendix B). Sequence alignments for each locus (nucLSU, nucSSU and mtSSU) and for each fungal class (Eurotiomycetes, Dothideomycetes, Leotiomycetes and Sordariomycetes) were prepared manually in BioEdit [68]. Introns and ambiguous SNPs were removed from the alignment. For a number of specimens we were unable to generate sequences for all of the selected loci and for other taxa sequences were not available in GenBank. The analyses included only samples with at least two sequenced loci. 

As the single locus analyses were congruent for the four individual classes, the final sequence analyses were performed on combined 3-locus datasets (nuLSU, nuSSU and mtSSU) for Dothideomycetes and Eurotiomycetes and 2-locus datasets (nuLSU and nuSSU) for Leotiomycetes and Sordariomycetes as in previous studies [9,69,70,71]. The multilocus datasets were generated with the SequenceMatrix program [72] and the phylogenetic analyses were performed using the maximum likelihood (ML) approach. In the multilocus datasets the loci were treated in partitions by genes nucLSU, nucSSU and mtSSU. The ML analyses were performed using the program RAxML v.7.1.3 [73], by applying the GTRMIX model. The phylogenetic trees were visualized in TreeView [74]. 

### 4.5. Statistical Analyses

Analyses and figures of media composition and growth preferences of fungal groups were performed in Microsoft Excel 2010 (Microsoft, Redmond, WA, USA) and CorelDrawX7.

## Figures and Tables

**Figure A1:**
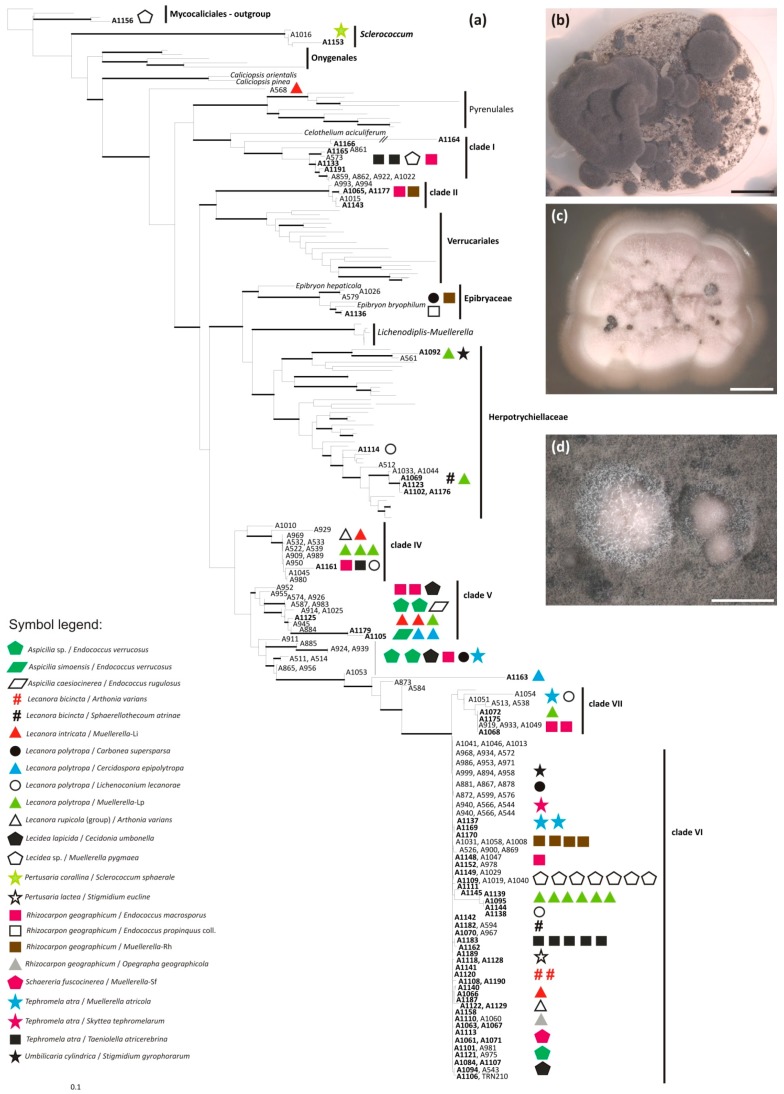
Phylogenetic position of fungal strains belonging to the class Eurotiomycetes (**a**) and habit of representative cultured strains: (**b**) A522 (clade IV); (**c**) A1133 (clade I); (**d**) A1175 (clade VII). The symbol legend describes the identity of the original samples from which the fungi were isolated: lichen species name/lichenicolous fungus species name infecting that lichen thallus. The isolated strains are reported with their DNA ID number; newly characterized strains are in bold; strains already characterized in Muggia et al. [9] and included in the analyses of growth media are not in bold. The nomenclature of the clades follows Muggia et al. [9]. Fully supported branches (bootstrap support = 100%) are in bold. Scale bar = (**b**,**c**) 4 mm, (**d**) 2 mm.

**Figure A2:**
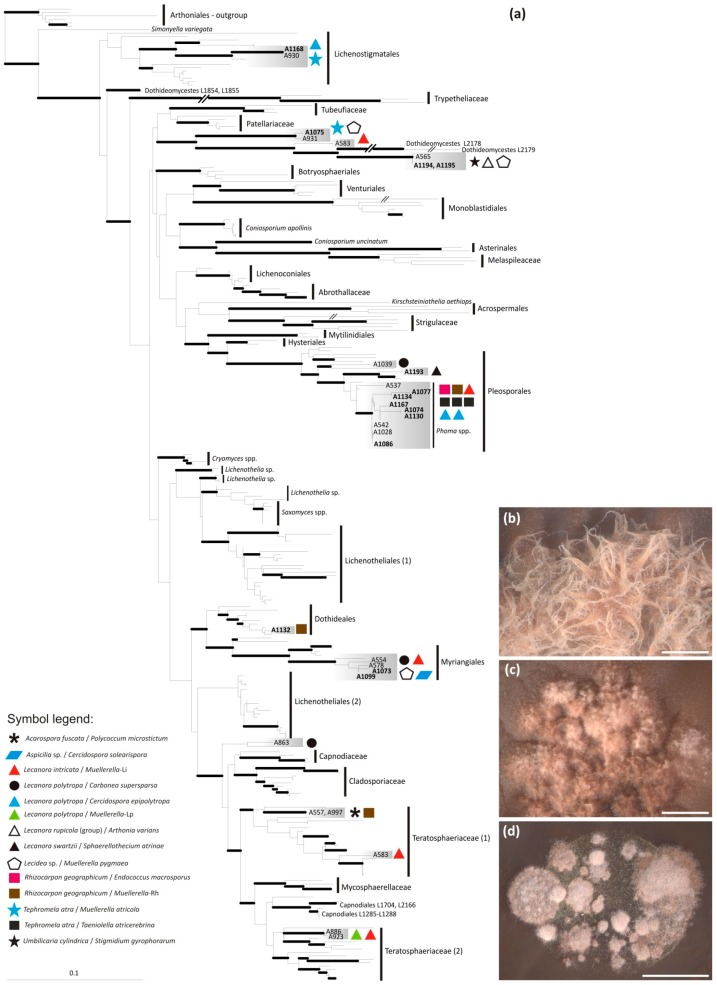
Phylogenetic position of fungal strains belonging to the class Dothideomycetes (**a**) and habit of representative cultured strains: (**b**) A1077 (Pleosporales, *Phoma* clade); (**c**) A1086 (Pleosporales, *Phoma* clade); (**d**) A1168 (Lichenostigmatales). The symbol legend reported describes the identity of the original samples from which the fungi were isolated: lichen species name/lichenicolous fungus species name infecting that lichen thallus. The isolated strains are reported with their DNA ID number; newly characterized strains are in bold; strains already characterized in Muggia et al. [9] and included in the analyses of growth media are not in bold. The nomenclature of the clades follows Muggia et al. [9]. Fully supported branches (bootstrap support = 100%) are in bold. Scale bar = (**b**) 2 mm, (**c**) 4 mm, (**d**) 5 mm.

**Figure A3:**
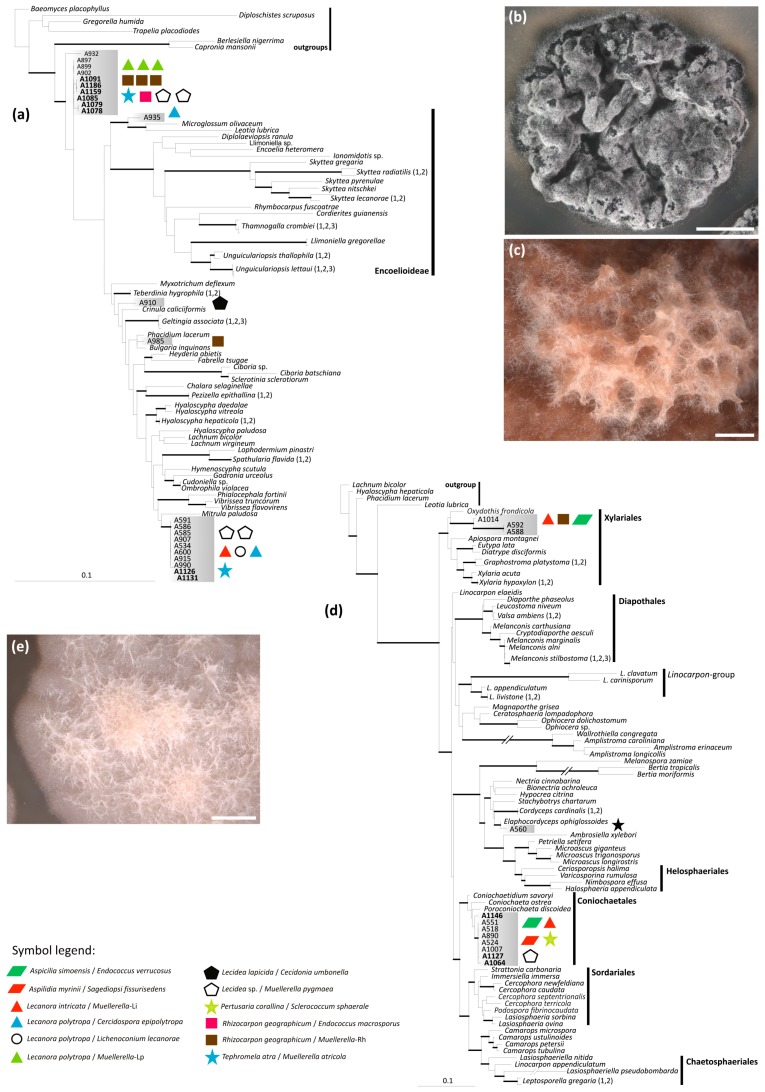
Phylogenetic position of fungal strains belonging to the class Leotiomycetes (**a**) and habit of representative cultured strains: (**b**) A1085 (Leotiomycetes) and (**c**) A1019 (Leotiomycetes). Phylogenetic position of fungal strains belonging to the class Sordariomycetes (**d**) and habit of representative cultured strain (**e**) A518 (Sordariomycetes, Coniochaetales). The symbol legend reported describes the identity of the original samples from which the fungi were isolated: lichen species name/lichenicolous fungus species name infecting that lichen thallus. The isolated strains are reported with their DNA ID number; newly characterized strains are in bold; strains already characterized in Muggia et al. [9] and included in the analyses of growth media are not in bold. The nomenclature of the clades follows Muggia et al. [9]. Fully supported branches (bootstrap support = 100%) are in bold. Scale bar = (**b**) 4 mm, (**c**) 1 mm, (**e**) 2 mm.

**Figure A4:**
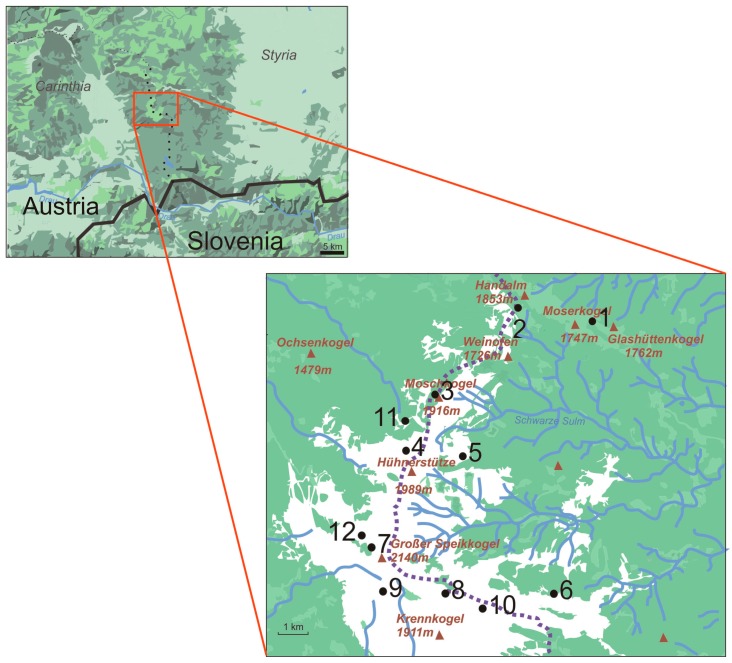
Schematic map of the geographic location of the collecting sites: the enlarged rectangle shows the detailed locations of the sites on the Koralpe mountain range.

**Figure A5:**
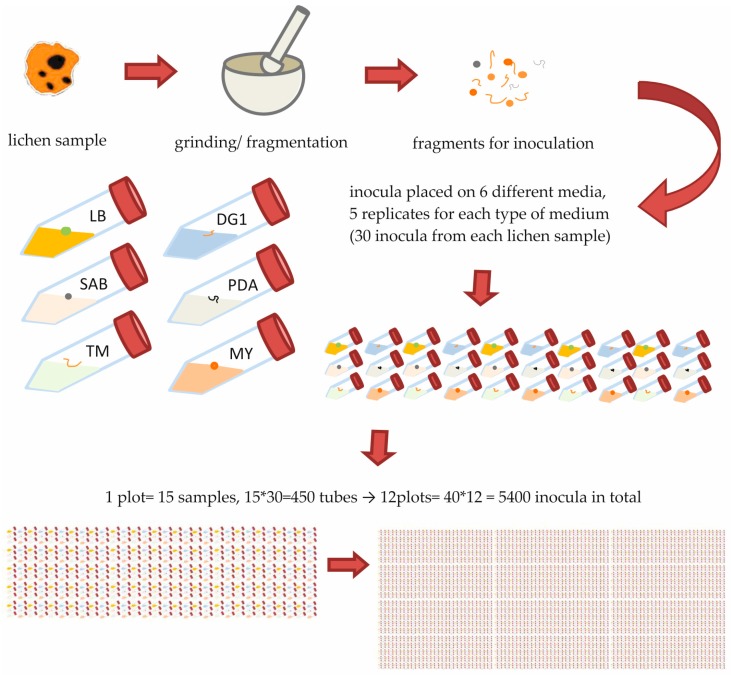
Workflow of the procedure used to set fungal isolates and replicates in the present research.

## Appendix A

**Table A1:** Lichens and lichenicolous fungal species and the number of thalli used in the culture experiments.

Lichen Species	N. of Thalli	Lichenicolous Fungus	N. of Thalli
*Acarospora fuscata*	1	*Polycoccum microsticticum*	1
*Aspicilia sinensis*	1	*Endococcus verrucosus*	1
*Aspicilia* sp.	8	*Cercidospora solearispora*	1
		*Endococcus rugulosus*	7
*Aspilidea myrinii*	13	*Sagediopsis fissurisedens*	13
*Immersaria athroocarpa*	1	*Muellerella pygmaea*-Ia *	1
*Lecanora bicincta*	9	*Arthonia varians*	7
		*Sphaerellothecium atryneae*	2
*Lecanora intricata*	5	*Muellerella pygmaea*-Li *	5
*Lecanora polytropa*	47	*Arthonia* sp.	3
		*Carbonea supersparsa*	3
		*Cercidospora epipolytropa*	14
		*Lichenoconium lecanorae*	11
		*Muellerella pygmaea*-Lp *	16
*Lecanora rupicola*	5	*Arthonia varans*	5
*Lecanora swartzii*	1	*Sphaerellothecium atryneae*	1
*Lecidea lapicida*	4	*Muellerella pygmaea*	4
*Lecidea* sp.	22	*Cecidonia umbonella*	2
		*Muellerella pygmaea var. pygmaea*	20
*Pertusaria corallina*	5	*Sclerococcum sphaerale*	5
*Pertusaria lactea*	4	*Stigmidium eucline*	4
*Protoparmelia badia*	1	*Phacographa protoparmeliae*	1
*Ophioparma ventosa*	1	*Muellerella pygm. var. ventosicola*	1
*Rhizocarpon geographicum* s.l.	36	*Endococcus macrosporus*	17
		*Endococcus propinquus*	1
		*Muellerella pygmaea*-Rg *	17
		*Opegrapha geographicola*	1
*Schaereria fuscocinerea*	2	*Endococcus perpusillus*	1
		*Muellerella pygmaea*	1
*Sporastatia polyspora*	1	*Polycoccum sporastatiae*	1
*Tephromela atra*	21	*Muellerella atricola*	3
		*Skyttea tephromelarum*	2
		*Taeniolella atricerebrina*	16
*Umbilicaria cylindrica*	2	*Stigmidium gyrophorarum*	2

(*) The lichenicolous fungus *Muellerella pygmaea* is reported with the initial of the species name of its host to differentiate the recognized varieties as not yet formally described [46].

## Appendix C

**Table A2:** NCBI accession numbers for the newly characterized isolates of lichen-inhabiting fungi included in the phylogenetic analyses of Appendix A.

DNA ID	NCBI Accessions		
**Eurotiomycetes**	**nuLSU**	**nuSSU**	**mtSSU**
A1061	MF071379	MF071329	MF085444
A1063	MF071380	MF071330	MF085445
A1065	MF071381	-	MF085446
A1066	MF071382	MF071331	MF085447
A1067	MF071383	MF071332	MF085448
A1068	MF071384	-	MF085449
A1069	MF071385	MF071333	-
A1070	MF071386	MF071334	MF085450
A1071	MF071387	MF071335	MF085451
A1072	MF071388	-	-
A1084	MF071389	MF071336	MF085452
A1092	MF071390	MF071337	-
A1094	MF071391	MF071338	MF085453
A1095	MF071392	-	MF085454
A1101	MF071393	-	MF085455
A1102	MF071394	-	MF085456
A1105	MF071395	-	MF085457
A1106	MF071396	MF071339	MF085458
A1107	MF071397	MF071340	-
A1108	MF071398	MF071341	MF085459
A1109	MF071399	MF071342	MF085460
A1110	MF071400	MF071343	-
A1111	MF071401	MF071344	MF085461
A1113	MF071402	MF071345	MF085462
A1114	MF071403	-	-
A1118	MF071404	MF071346	MF085463
A1120	MF071405	MF071347	MF085464
A1121	MF071406	MF071348	MF085465
A1122	MF071407	-	MF085466
A1123	MF071408	MF071349	MF085467
A1125	MF071409	MF071350	MF085468
A1128	MF071410	-	MF085469
A1129	MF071411	-	MF085470
A1133	MF071412	MF071351	MF085471
A1136	MF071413	-	MF085472
A1137	MF071414	MF071352	MF085473
A1138	MF071415	-	MF085474
A1139	MF071416	-	MF085475
A1140	MF071417	-	MF085476
A1141	MF071418	-	MF085477
A1142	-	MF071353	MF085478
A1143	MF071419	-	MF085479
A1144	MF071420	MF071354	MF085480
A1145	MF071421	MF071355	MF085481
A1148	MF071422	-	MF085482
A1149	MF071423	-	MF085483
A1152	MF071424	-	MF085484
A1153	MF071425	-	MF085485
A1156	-	MF071356	MF085486
A1158	MF071426	-	MF085487
A1161	MF071427	-	MF085488
A1162	MF071428	-	-
A1163	MF071429	-	MF085489
A1164	MF071430	-	MF085490
A1165	-	MF071357	MF0854910
A1166	MF071431	-	MF085492
A1169	MF071432	-	MF085493
A1170	MF071433	-	MF085494
A1175	-	-	MF085495
A1176	-	-	MF085496
A1177	MF071434	-	MF085497
A1179	MF071435	-	MF085498
A1182	MF071436	-	MF085499
A1183	MF071437	-	MF085500
A1187	MF071438	-	MF085501
A1189	MF071439	-	MF085502
A1190	MF071440	-	-
A1191	-	MF071358	MF085503
**Dothideomycetes**	**nuLSU**	**nuSSU**	**mtSSU**
A1073	MF071367	MF071322	-
A1074	MF071368	MF071323	MF085504
A1075	-	MF071324	MF085505
A1077	MF071369	MF071325	MF085506
A1086	MF071370	-	MF085507
A1099	MF071371	MF071326	MF085508
A1130	MF071372	-	MF085509
A1132	MF071373	-	-
A1134	MF071374	-	MF085510
A1167	-	MF071327	MF085511
A1168	MF071375	MF071328	-
A1193	MF071376	-	MF085512
A1194	MF071377	-	MF085513
A1195	MF071378	-	MF085514
**Leotiomycetes**	**nuLSU**	**nuSSU**	**mtSSU**
A1078	MF071361	MF071314	MF085515
A1079	MF071362	MF071315	MF085516
A1085	MF071363	MF071316	MF085517
A1091	MF071364	MF071317	MF085518
A1126	MF071365	MF071318	-
A1131	MF071366	MF071319	-
A1159	-	MF071320	MF085519
A1186	-	MF071321	MF085520
**Sordariomycetes**	**nuLSU**	**nuSSU**	**mtSSU**
A1064	-	MF071311	MF085521
A1127	MF071360	MF071312	MF085522
A1146	MF071359	MF071313	MF085523

## Appendix D

Composition of the growth media used to isolate and culture lichen-associated fungi and lichen mycobionts.

**Table A3:** Lilly & Barnett medium (LB, Lilly and Barnett, 1951).

Nutrients	Total Amount of Nutrient in 1 L Medium
Glucose	10 g
Asparagine	2 g
K_2_HPO_4_	1 g
MgSO_4_·7H_2_O	0.5 g
Fe(NO_3_)_3_·9H_2_O	0.2 g
ZnSO_4_·7H_2_O	0.2 g
MnSO_4_·4H_2_O	0.1 g
Thiamine	100 µg (stock solution: 100 mg/L), 1 mL from the stock solution
Biotin	5 µg (stock solution: 25 mg/L) 200 µL from the stock solution
Agar	15 g
Distilled H_2_O	986 mL

**Table A4:** *Trebouxia* medium (TM, Ahmadjian, 1987).

Nutrients	Total Amount of Nutrient in 1 L Medium
BBM	970 mL
Peptone	10 g
Glucose	20 g
Agar	20 g

**Table A5:** Bold’s Basal medium (BBM, Nichols and Bold, 1965) as part of the TM components.

Solutions A1 ^a^		400 mL	Solutions A2		1000 mL
A1/1	NaNO_3_	10 g	A2/1	H_3_BO_3_	11.42 g/L
A1/2	KH_2_PO_4_	7 g	A2/2	FeSO_4_·7H_2_O ZnSO_4_·7H_2_O MnCl_2_·4H_2_O	4.98 g/L 8.82 g/L 1.44 g/L
A1/3	K_2_HPO_4_	3 g	A2/3	MoO_3_ CuSO_4_·5H_2_O Co(NO_3_)_3_·6H_2_O	0.7 1g/L 1.57 g/L 0.49 g/L
A1/4	MgSO_4_·7H_2_O	3 g	A2/4	EDTA KOH	50 g/L 31 g/L
A1/5	CaCl	1 g			
A1/6	NaCl	1 g			

**^a^** 10 mL of each solutions A1/1–A1/6 and 1 mL of each solution A2/1–A2/4 are added into 1000 mL distilled water.

**Table A6:** Malt Yeast-extract (MY, Lilly and Barnett, 1951).

Nutrients	Total Amount of Nutrient in 1 L Medium
Malt extract	20 g
Yeast extract	2 g
Agar	20 g

**Table A7:** Sabouraud (SAB, Pagano, Levin and Trejo, 1958).

Nutrients	Total Amount of Nutrient in 1 L Medium
Glucose	20 g
Peptone	10 g
Yeast extract	5 g
Agar	20 g

**Table A8:** Potato Dextrose agar (PDA, ApplChem, A5828, pH 5.6 (20 °C).

Nutrients	Total Amount of Nutrient in 1 L Medium
Potato extract (solid)	4 g
Glucose	20 g
Agar	15 g

**Table A9:** Dichloran/Glycerol agar (DG18, Hocking and Pitt, 1980 pH: 5.6 ± 0.2 at 25 °C).

Nutrients	Total Amount of Nutrient in 1 L Medium
Peptone	5 g
Glucose	10 g
Potassium dihydrogen phosphate	1 g
Dichloran 0.2% in EtOH 1 mL	0.002 g
Magnesium sulfate	0.5 g
Chloramphenicol	0.1 g
Agar	15.0 g

## Appendix E

**Table A10:** Geographic information of the collecting sites is reported: ID number of the site, name of the site, geographical coordinates and altitude. All sites are located in subalpine to alpine habitat above the timberline, ranging between 1800 and 2100 m above sea level (a.s.l.), on the Koralpe Mountain range, in the region Styria (ST) and Carinthia (K) in Austria. Landscape characterized by big boulders and cliffs of homogeneous size of siliceous-schist/gneissic rocks separated by wide areas of pastures or dwarf shrub formations.

Site N.	Mountain Top Name	Latitude	Longitude	Altitude a.s.l.
1	Glashüttenkogel (ST)	46°50′12′′ N–46°50′′20′′ N	15°02′35′′ E–15°03′00′′ E	1750 m
2	Handalm (ST)	46°50′38′′ N	15°01′10′′ E	1800 m
3	Moschkogel (ST)	46°49′21′′ N–46°49′22′′ N	14°59′29′′ E–14°59′34′′ E	1900 m
4	Hühnerstütze (K)	46°48′23′′ N–46°48′31′′ N	14°58′57′′ E–14°59′08′′ E	1970 m
5	Loskogel (ST)	46°48′23′′ N–46°48′25′′ N	15°00′31′′ E–15°00′33′′ E	1780 m
6	Glitzfelsen (ST)	46°46′50′′ N–46°46′53′′ N	15°01′24′′ E–15°01′35′′ E	1810 m
7	Großer Speikkogel (K)	46°47′21′′ N–46°47′23′′ N	14°57′58′′ E–14°58′06′′ E	2088 m
8	Ochsenstein (K)	46°46′51′′ N–46°46′54′′ N	14°59′23′′ E–14°59′26′′ E	1980 m
9	Krakaberg (K)	46°46′43′′ N–46°46′47′′ N	14°58′13′′ E–14°58′14′′ E	2050 m
10	Ochsenofen (K)	46°46′29′′ N–46°46′31′′ N	15°00′45′′ E–15°00′52′′ E	1760 m
11	Großer Speikkogel (K)	46°47′39′′ N	14°57′42′′ E	2000 m
12	Sprungkogel (K)	46°48′54′′ N	14°58′14′′ E	1860 m
